# 

**Published:** 1899-01

**Authors:** 


### PERSONALS.

Dr. Nelson P. Hinkley, of Buffalo, N. Y., has severed by resignation his connection with the Bureau of Animal Industry, and has also leased his hospital in Buffalo to Drs. Baker and Ryder.

Dr. William H. Pendry, of the Borough of Brooklyn, has been appointed lecturer on veterinary jurisprudence at the American Veterinary College.

Dr. R. S. Huidekoper made a flying visit to New York City in December.

Dr. C. H. Zink, of Kansas City, has been transferred to the port of Buffalo, succeeding to the position of Dr. Nelson P. Hinkley.

Dr. H. D. Gill, dean of the New York College of Veterinary Surgeons, was special referee for the firm of William B. Fasig & Co. at the recent two weeks' sale in Greater New York.

Dr. William H. Pendry, of the American Veterinary College Alumni Association, was a strong advocate of holding the meeting of the American Veterinary Medical Association in New York City.

Dr. E. G. Gilbert, of Pottstown, Pa., was a visitor to the JOURNAL office in the early part of December.

Mr. R. A. Pearson, of the dairy division, Department of Agriculture, Washington, D. C., spent some two weeks in North Dakota in December.

Drs. A. D. Melvin and V. A. Norgaard, of the Bureau of Animal Industry, have been assigned to duty at Tampa, Fla., to investigate the extent of glanders among army horses.

Drs. M. S. Lantz and E. S. Walmer, of the United States College of Veterinary Surgeons, sent South by the army department, will include Jacksonville and Tampa among their inspection work.

Dr. C. P. Lyman, of Boston, presented an interesting and instructive address at the college opening of Harvard's veterinary department in October.

Dr. C. D. Smead, of Logan, N. Y., fills the post of secretary to the New York State Shropshire Breeders' Association.

Dr. E. A. Gruver, of Richlandtown, Pa, graduate of the New York College of Veterinary Surgeons, class of 1894, was a recent visitor to the JOURNAL office.

Dr. W. Horace Hoskins, editor of the JOURNAL, addressed the Woman's Health Protective Association in November on "Municipal Meat Inpection" and Philadelphia's needs in this direction.

Dr. Leonard Pearson, of Philadelphia, was a visitor to Williamsport in December. An interesting article contributed to the Dairymen's Union of Pennsylvania has just been issued from his pen.

Dr. D. E. Salmon, chief of the Bureau of Animal Industry, contributed a series of articles on Texas fever to the *Breeders' Gazette* during December.

Dr. H. M. Burgess, of the Bureau of Animal Industry, has been transferred from St. Joseph, Mo., to Louisville, Ky.

Dr. S. L. Gelston, assistant surgeon of the United States College of Veterinary Surgeons, Washington, D. C., was a visitor to the JOURNAL office in December.

The many friends of Dr. F. H. Osgood, of Boston, Mass., will regret to learn of his misfortune in December in the loss of his second son, fifteen years of age, from the sequelæ of diphtheria.

Dr. R. S. Huidekoper spent the Christmas holidays among his many friends in the "City of Brotherly Love."

Professor A. Smith, of Toronto, Canada, will preside over the duties of the Executive Committee of the Ontario Jockey Club the coming year.

Dr. J. J. Drasky, of Crete, Neb., takes an active interest in the Nebraska Jersey Breeders' Association and fills a place on the executive committee of that organization.

Dr. Theobald Smith, of Boston, addressed the Maine Dairymen's Association in December on "Sanitary Aspects of Dairying."

Dr. Thomas B. Rayner, of Chestnut Hill, Philadelphia, has been seriously ill since the loss of his daughter in December.

Dr. Louis O. Lusson, of Ardmore, Pa., was a victim of the "grippe" in December and confined to the house for some three weeks.

Dr. F. H. P. Edwards, of Iowa City, addressed the Iowa Improved Stock Breeders' Association in December on "The Principal Diseases that are Intercommunicable Between Man and Animals."

Dr. S. J. J. Harger, of Philadelphia, contributed an article on "Horse Breeding" to the December 7th issue of the *Breeders' Gazette.*

Mr. R. A. Pearson, assistant chief of the dairy division of the Department of Agriculture, addressed the Washington State Dairymen's Association in December.

Dr. W. Horace Hoskins, of the JOURNAL'S staff, was selected chairman of the convention to revise the rules of the Democratic party in the city of Philadelphia in December.

State Veterinarian Leonard Pearson, of Philadelphia, was an expert witness in the Dauphin and Chester County Courts in December.

Dr. A. B. Campbell, of Berlin, Ontario, has added to his practice an established livery stable and blacksmith shop, which he will direct in connection with his professional work.

"A Lost Sheep Found" is the title of an article in the Christmas number of the *Breeders' Gazette*, by Dr. A. S. Alexander, of Evanston, Ill.

Drs. Albert Long, of Lewiston, Me., and H. D. Fenimore, of Knoxville, Tenn., have been added to the corps of inspectors of the Bureau of Animal Industry at Kansas City, Kan.

Dr. James S. Mecray, of Maple Shade, N. J., is an ardent sportsman, and follows the gun in the field with all the ardor of a mighty Nimrod.

Veterinarian Edward Roswell Newton, of Boston, has had the degree of M.D. added to his name, graduating in the medical class of Harvard in June.

Dr. Jos. B. Clancy, of Chicago, Inspector of the Bureau of Animal Industry, is taking a special course in laboratory work.

Dr. A. C. Van Ness, of Denver, Col., fills the post of veterinarian to the Fire and Police Departments of that city.

Dr. George Townsend, of New Glasgow, Nova Scotia, holds the position of inspector of livestock for that district.

Dr. Leonard Pearson, State Veterinarian, and M. E. Conard, of West Grove, Pa., were among the speakers at the Farmers' Institute at Pottstown in December.

Dr. William H. Ridge, of Trevose, Pa., and J. Curtis Michener, of Colmar, Pa., addressed in January the Farmers' Institute on the "Value of Corn for Feeding" and "The Present and Future Status of Agriculture."

Dr. William H. Pendry, of the Borough of Brooklyn, is an active worker in the Royal Arcanum, and fills a high official position.

Dr. John J. Repp, of Philadelphia, representing the State Livestock Sanitary Board, visited the western part of Franklin County, Pa., in December to examine into a reported extensive and very fatal outbreak of anthrax covering some twenty farms.

Drs. John W. Adams, S. J. J. Harger, William G. Shaw, and W. Horace Hoskins were present as guests at the banquet of the Students' Society of the Veterinary Department of the University of Pennsylvania on December 16, 1898.

Dr. John J. Repp, of Philadelphia, Pa., received in December the appointment to the chair of bacteriology by the Board of Regents of the Iowa State Agricultural College and Mechanic Arts.

Professor Wesley Mills, of the Veterinary Department of the McGill University, Montreal, has returned from abroad and resumed his work as an instructor in the above institution.

Dr. A. L. Compton, of Perry, Mich., had the misfortune of having lost by fire in November the entire outfit of office, pharmacy, and library.

Dr. J. E. Spindler, of Tyrone, Pa., addressed the Huntingdon County Farmers' Institute in December on "Diseases of the Dairy Cow."

Dr. Harry Walters and wife, of Wilkesbarre, after six weeks of fighting the "grippe" complicated with malaria, left Philadelphia this month for a sojourn in southern California.

Dr. E. T. Hagyard, veterinarian to the Marcus Daly Bitter Root Farm, in Montana, recently attended a shipment of broodmares to England.

Dr. W. G. Huyett, of Wernersville, Pa., has removed to Chicago, Ill.

Dr. A. F. Schrieber, of Philadelphia, is an ardent admirer of the pit game variety of poultry, and in his numerous pens has many fine specimens of these pugilistic birds.

Dr. A. E. Gruver, of Richlandtown, Pa., graduate of the New York College of Veterinary Surgeons, class of 1894, was a recent visitor to the JOURNAL office.

Dr. R. P. Linehart, of Wayne, Pa., reports an extensive outbreak of canine rabies in that district, with one child bitten, who has been sent to the Pasteur Institute at New York for treatment.

---

Secretary Wilson, of the Department of Agriculture, strongly urges better salaries for the heads of divisions and departments. He points to the constant losses suffered by the department in equipping men specially for certain lines of work and then losing them to other institutions and organizations because of the inadequate salaries given. He urges the securing of the best men from agricultural schools and colleges by civil-service examination, and the training of these men for these positions by placing them as assistants to the heads of departments.

"Old Ned," at forty-three years of age, of civil-war fame, died at North Easton, Pa., in May.

